# Immune Checkpoint Inhibitors Beyond Progression in Various Solid Tumors: A Systematic Review and Pooled Analysis

**DOI:** 10.3390/jcm14186680

**Published:** 2025-09-22

**Authors:** Fausto Petrelli, Antonio Ghidini, Maria Chiara Parati, Karen Borgonovo, Mauro Rossitto, Mara Ghilardi, Giuseppina Dognini, Daniela Petro’, Irene Angeli, Veronica Lonati, Lorenzo Dottorini, Alessandro Iaculli

**Affiliations:** 1 Oncology Unit, ASST Bergamo Ovest, 24047 Treviglio, BG, Italy; mariachiara_parati@asst-bgovest.it (M.C.P.); karen_borgonovo@asst-bgovest.it (K.B.); mauro_rossitto@asst-bgovest.it (M.R.); mara_ghilardi@asst-bgovest.it (M.G.); giuseppina_dognini@asst-bgovest.it (G.D.); daniela_petro@asst-bgovest.it (D.P.); irene_angeli@asst-bgovest.it (I.A.); veronica_lonati@asst-bgovest.it (V.L.); lorenzo_dottorini@asst-bgovest.it (L.D.); 2 Oncology Unit, Casa di Cura Igea, 20100 Milan, Italy; antonioghidini@hotmail.com; 3 Oncology Unit, ASST Bergamo Est, 24068 Seriate, BG, Italy; alessandro.iaculli@asst-bergamoest.it

**Keywords:** immune checkpoint inhibitors, cancer, beyond progression, systematic review

## Abstract

**Background**: Immune checkpoint inhibitors (ICIs) have transformed outcomes in advanced cancers; however, the value of continuing treatment after radiologic progression remains uncertain. We systematically assessed the efficacy and safety of ICI continuation beyond progression, focusing on the objective response rate (ORR), progression-free survival (PFS), and overall survival (OS). **Methods:** PubMed/MEDLINE, Embase, and the Cochrane Library were searched from inception to 31 March 2025. Eligible reports included retrospective cohorts, prospective trials, post hoc analyses, and pooled regulatory reviews that compared outcomes after ICI continuation versus discontinuation or historical controls. Quality was appraised with the Newcastle–Ottawa Scale (observational designs) and the Cochrane Risk-of-Bias tool (randomized trials). **Results:** Fifty studies involving 8989 patients met the inclusion criteria: 41 retrospective cohorts; 6 post hoc analyses; 2 randomized trials (1 phase III, 1 phase II); and 1 pooled FDA review. Continuing ICIs beyond progression produced ORRs of 9.3–39% in non-small cell lung cancer (n = 5102), 14–100% in melanoma (n = 669), and 8–33% in renal cell carcinoma (n = 458). Median OS ranged from 8.9 to 18.2 months in lung cancer, 12 to 29.9 months in melanoma, and up to 34.8 months in RCC. Modest but clinically meaningful benefits were reported in colorectal, head-and-neck, gastric, liver, and urothelial tumors. **Conclusions:** Select patients—particularly those with melanoma, lung cancer, RCC, or gastric cancer—may derive sustained benefit from ICI therapy after radiologic progression. Decisions should incorporate tumor biology, performance status, and emerging biomarkers. Prospective, biomarker-driven trials are needed to define optimal patient selection and the duration of post-progression immunotherapy.

## 1. Introduction

Immune checkpoint inhibitors (ICIs) have irreversibly altered the natural history of a wide spectrum of advanced malignancies, ushering in an era in which durable remissions and long-term survival are achievable outcomes for a clinically meaningful minority of patients. Agents targeting programmed death-1 (PD-1) and its ligand (PD-L1), cytotoxic T-lymphocyte–associated antigen 4 (CTLA-4), and—more recently—next-generation checkpoints, such as lymphocyte activation gene-3 (LAG-3), T-cell immunoreceptor with Ig and ITIM domains (TIGIT), and B and T lymphocyte attenuator (BTLA), have demonstrated the capacity to unshackle anti-tumor immunity and to reshape therapeutic algorithms across melanoma, non-small cell lung cancer (NSCLC), renal cell carcinoma (RCC), and numerous other tumor types. Despite these advances, disease progression—defined radiologically by Response Evaluation Criteria in Solid Tumors (RECIST) v1.1—occurs in 40–70% of patients within the first year of therapy, even in the setting of an initial partial response or disease stabilization. Crucially, conventional response criteria were designed for cytotoxic agents that exert direct tumor cytostasis or cytolysis; they are ill-equipped to capture the unique kinetic patterns that emerge when tumor regression is mediated indirectly by host immunity. Atypical phenomena, such as pseudoprogression, delayed responses, and hyperprogression, challenge the doctrinal linkage between radiographic growth and therapeutic futility. Recognizing these nuances, immune-adapted frameworks—including immune-related response criteria, iRECIST, and imRECIST—have been proposed; however, they remain inconsistently adopted in daily practice [1,2,3,4].

Treatment beyond progression (TBP) rests on two conceptual pillars. First, enlargement of target lesions or the appearance of new lesions may not invariably denote biological escape. Instead, radiographic growth can reflect transient lymphocytic infiltration, oedema, or necrotic remodeling—morphological surrogates of an evolving, productive immune response. Second, even genuine clonal outgrowth in one anatomical compartment (oligoprogression) does not preclude ongoing immune control at other sites. Continuing ICIs may therefore preserve or reinvigorate systemic immunity, while focal modalities, such as stereotactic ablative radiotherapy, eradicate resistant subclones [5,6,7].

Pre-clinical models corroborate this paradigm: sustained PD-1 blockade maintains a pool of tumor-reactive T cells exhibiting memory and stem-like phenotypes that are capable of mounting renewed effector responses upon antigenic re-stimulation. Interrupting the checkpoint signal prematurely can trigger T-cell exhaustion, contraction of the memory pool, and resurgent tumor growth. Clinically, longitudinal biopsies and peripheral blood analyses demonstrate persistent expansion of PD-1^high CD8^+^ T-cell clones and heightened interferon-γ gene signatures in patients who remain on therapy after apparent progression, whereas these immune correlates wane in those who discontinue early [8,9].

Large-scale retrospective series encompassing nearly 9000 patients revealed that 9–39% of NSCLC patients, 14–100% of melanoma cases, and up to one-third of RCC patients treated beyond RECIST progression ultimately achieved further tumor shrinkage or durable disease stabilization. Among the remainder, a distinct subgroup experienced indolent disease kinetics—characterized by a tumor doubling time exceeding six months, limited symptom burden, and preserved performance status—arguably benefiting from ongoing immune surveillance, even in the absence of radiographic responses [10,11,12,13,14,15,16,17].

By providing an integrated perspective, we aim to inform evidence-based selection of patients most likely to gain incremental benefit from TBP, thereby maximizing therapeutic value while stewarding healthcare resources.

## 2. Material and Methods

### 2.1. Search Strategy and Eligibility Criteria

We adhered to PRISMA 2020 guidelines. A medical librarian designed comprehensive queries for PubMed/MEDLINE, Embase, Web of Science Core Collection, and the Cochrane Central Register of Controlled Trials, covering inception to 31 March 2025, with alerts activated to capture articles published during manuscript preparation. We combined MeSH/Emtree terms and free-text synonyms for immune checkpoint inhibition (e.g., “CTLA-4 blockade,” “PD-1 inhibitors,” “LAG-3 antibody”) with terms describing radiological progression (“RECIST,” “pseudoprogression,” “treatment beyond progression,” “rechallenge”). ClinicalTrials.gov, the WHO International Clinical Trials Registry Platform, and major oncology congress abstract books (ASCO, ESMO, AACR, SITC) from 2015–2024 were searched for unpublished or ongoing studies. Only peer-reviewed full articles in English that enrolled ≥10 adults with advanced solid tumors treated with ICIs (single agents or combinations) beyond RECIST progression and reported at least one efficacy endpoint were eligible. Case reports, pediatric series, trials halted for toxicity, non-oncology indications, studies with experimental drugs still not available in clinical practice, and duplicate datasets were excluded.

### 2.2. Study Selection, Data Extraction, and Quality Appraisal

Two reviewers (FP, AG) independently screened titles/abstracts, and, subsequently, full texts; disagreements were reconciled by consensus with a third senior reviewer (AI). Extraction employed a piloted, standardized form capturing study design; population characteristics; tumor histotype; checkpoint class; line and duration of therapy; definition of progression; and local ablative interventions and outcomes (objective response rate [ORR]; disease-control rate progression-free survival [PFS], overall survival [OS], and immune-related adverse events). When hazard ratios were not reported, they were reconstructed from Kaplan–Meier curves using the Parmar algorithm.

Methodological quality for the randomized trials was assessed with Cochrane RoB 2, while observational cohorts were appraised using the Newcastle–Ottawa Scale (NOS). Inter-rater agreement was quantified with Cohen’s κ; κ > 0.80 was deemed excellent.

### 2.3. Data Synthesis and Statistical Analysis

This analysis employed a random-effects meta-analysis approach to pool hazard ratios across the studies that reported them. The inverse variance method was used to weight studies according to the precision of their effect estimates. For studies that did not report hazard ratios but provided sufficient data on survival outcomes, HRs were estimated using the methods described by Tierney et al.

The synthesis process involved the following steps:

Hazard Ratio Pooling: HRs for PFS and OS were pooled separately across studies using a random-effects model. Studies were weighted according to the inverse of the variance of the log hazard ratio.

1. Heterogeneity Assessment: Statistical heterogeneity was assessed using the I^2^ statistic, with values of 25%, 50%, and 75% considered to represent low, moderate, and high heterogeneity, respectively;

2. Subgroup Analysis: Where possible, separate pooled estimates were calculated for specific cancer types (e.g., melanoma, nasopharyngeal carcinoma) to explore potential differences in treatment effect across tumor types;

3. Sensitivity Analysis: To assess the robustness of the findings, sensitivity analyses were conducted by sequentially excluding each study from the pooled estimate.

Pooled hazard ratios with 95% confidence intervals are reported for both PFS and OS outcomes, providing a quantitative estimate of the overall treatment effect of TBP or rechallenge strategies compared to alternative approaches.

**Figure 1:**
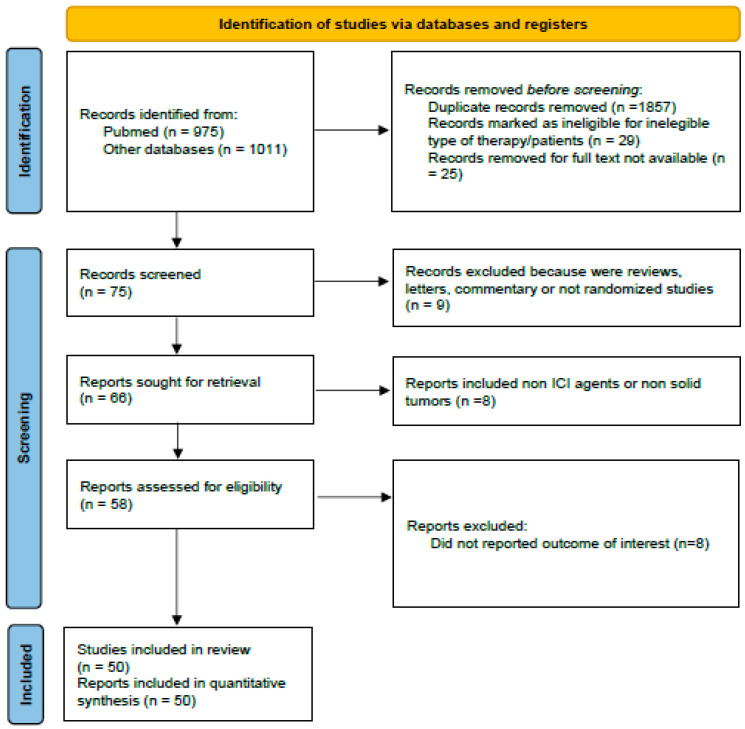
Flow diagram of included studies.

All computations were executed in R v4.3.2 using the ‘meta’ and ‘metafor’ packages. A detailed PRISMA 2020 flow diagram of study selection is provided in Figure 1.

## 3. Results

### 3.1. Literature Search and Study Selection

The literature review identified 50 studies evaluating immune checkpoint inhibitors (ICIs) beyond radiological progression in various cancers, including lung cancer, melanoma, renal cell carcinoma (RCC), colorectal cancer (CRC), head-and-neck, liver, gastric, and urothelial cancers [10,11,12,13,18,19,20,21,22,23,24,25,26,27,28,29,30,31,32,33,34,35,36,37,38,39,40,41,42,43,44,45,46,47,48,49,50,51,52,53,54,55,56,57,58,59,60,61,62,63]. Following title, abstract, and full-text screening, 41 were retrospective cohorts, 6 were post hoc clinical trial analyses, 1 was a pooled FDA analysis, and 2 were prospective trials. Collectively, these studies enrolled 8989 patients: 5102 with lung cancer, 1164 with melanoma, 566 with RCC, and 2157 with other cancers.

Most retrospective studies showed moderate quality, often limited by selection bias and population heterogeneity. Higher-quality data were obtained from prospective studies and pooled analyses, although these were fewer and involved smaller cohorts. Screening criteria are detailed in the PRISMA diagram (Figure 1).

### 3.2. Quality Assessment of Included Studies

Among the 29 studies involving lung cancer, 27 were retrospective (moderate quality: Newcastle–Ottawa Scale scores 5–7). Two were post hoc RCT analyses with a moderate risk of bias. For melanoma, six retrospective studies were also scored as moderate, with one FDA pooled analysis and one phase 3 trial contributing higher-quality evidence. For RCC, five were retrospective and two were post hoc trials, all moderate in quality. One study (Murianni et al.) [64] had high-quality reporting.

Mixed cancer studies (n = 18) were largely retrospective (moderate quality). A phase 2 trial (Hirano et al.) and a phase 3 trial (Boku et al.) provided higher-quality evidence. Overall, evidence ranged from moderate to low quality, due to retrospective design, variable patient selection, and inconsistent outcome metrics. Nonetheless, select high-quality studies enhanced the evidence for melanoma, gastric cancer, and NSCLC. These findings highlight the need for standardized, prospective trials.

### 3.3. Definition of Progression

Radiologic progression was recorded as defined in each primary study.

- RECIST v1.1 was the dominant standard: 41/50 studies (82%)—including 27/29 lung cancer cohorts and all but 1 RCC series—declared progression exclusively by RECIST v1.1;
- Immune-adapted criteria were used in six studies (12%): four trial or post hoc datasets applied iRECIST and two melanoma cohorts used immune-related or immunomodified RECIST (irRC/imRECIST) to mandate confirmatory scans after an initial ≥20% tumor-burden increase.

The remaining three observational series relied on investigator assessment when formal criteria were not stated.

Across the 50 studies, progression definitions were largely homogeneous: 82% used RECIST v1.1, while 12% adopted immune-adapted frameworks that required confirmation to avoid misclassifying atypical responses. Three key observations emerged:

Incidence of pseudoprogression—when explicitly reported—ranged from 2–7% in NSCLC cohorts to 15–20% in melanoma studies and was <2% in microsatellite-stable colorectal cancer.

All studies that used iRECIST or irRC permitted treatment continuation while awaiting a confirmatory scan; conversely, RECIST-only series counted the first progressive scan as the event but often still allowed for treatment beyond progression at the physician’s discretion.

Where described, pseudoprogression was operationalized as an initial ≥ 20% increase (or new lesions) followed by ≥30% tumor shrinkage or stabilization on the next imaging 4–12 weeks later, in the absence of clinical deterioration.

### 3.4. Treatment Efficacy

#### 3.4.1. Lung Cancer (Table 1, Table 2 and Table 3)

**Figure 2:**
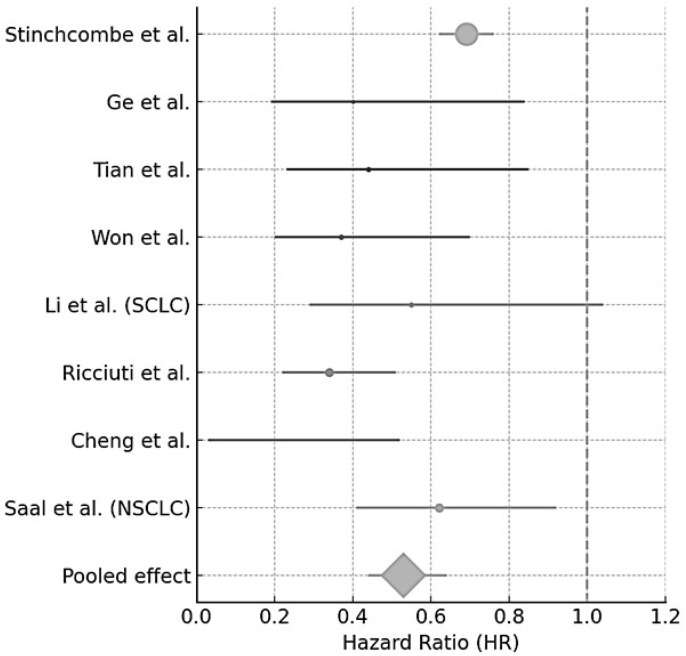
Forest plot for OS in lung cancer studies [19,23,25,28,29,32,33,38].

Of the 5102 lung cancer patients across 29 studies, most received ICIs beyond progression via retrospective protocols. Objective response rates (ORRs) ranged from 10.3% to 46.6%. Median progression-free survival (PFS) varied from 0.5 to 12.2 months, with most between 3 and 6 months. OS spanned 2.8 to 31.5 months and was highest in patients with initial durable responses. Factors associated with better outcomes included early response, lack of metastases, and treatment intensification (e.g., ICI switches). Only two reports provided data for SCLC, resulting in very few data from which to draw conclusions. A meta-analysis of studies with HRs available was attempted and is shown in Figure 2.

**Table 1:** Lung cancers: OS.

Study	Hazard Ratio for OS (95% CI)	Weight (%)
Stinchcombe et al. [32]	0.69 (0.62–0.76)	28.5
Ge et al. [23]	0.40 (0.19–0.84)	5.2
Tian et al. [33]	0.44 (0.23–0.85)	6.3
Won et al. [38]	0.37 (0.20–0.70)	6.6
Li et al. (SCLC) [25]	0.55 (0.29–1.04)	6.4
Ricciuti et al. [28]	0.34 (0.22–0.51)	10.8
Cheng et al. [19]	0.13 (0.03–0.52)	1.7
Saal et al. (NSCLC) [29]	0.62 (0.41–0.92)	11.2
Pooled HR	0.53 (0.44–0.64)	

**Table 2:** Overall survival by subgroups.

Subgroup		Hazard Ratio (HR)	95% CI
Overall NSCLC		0.45	0.32–0.63
Squamous NSCLC		0.51	0.32–0.81
Non-squamous NSCLC		0.42	0.28–0.63
Driver gene–negative NSCLC		0.13	0.01–0.74
Driver gene–positive NSCLC		0.68	0.42–1.10
Extensive-stage SCLC		0.39	0.16–0.92
**Category**	**Subgroup**	**HR**	**95% CI**
**PD-L1 expression**	High (≥50%)	0.32	0.18–0.57
	Intermediate (1–49%)	0.47	0.29–0.76
	Negative (<1%)	0.61	0.39–0.95
**Treatment line**	First-line	0.39	0.18–0.84
	Second-line	0.44	0.29–0.67
	Third-line or later	0.51	0.32–0.81
**Pattern of progression**	Slow progression (≤20% increase)	0.31	0.18–0.53
	Rapid progression (>20% increase)	0.62	0.41–0.94
	New lesions only	0.45	0.28–0.72
	Target lesion growth + new lesions	0.58	0.37–0.91
**Duration of TBP**	<6 weeks	0.65	0.43–0.98
	6–12 weeks	0.42	0.27–0.65
	>12 weeks	0.29	0.17–0.49

**Table 3:** Several studies reported on PFS2 (time from initial progression to second progression or death).

Study	Median PFS2 for TBP	Median PFS2 for non-TBP	HR (95% CI)
Cheng et al. [19]	4.6 months	2.1 months	0.51 (0.34–0.76)
Guven et al. [24]	3.8 months	1.9 months	0.48 (0.33–0.69)
Li et al. [55]	4.2 months	2.3 months	0.45 (0.21–0.97)
Yin et al. [43]	4.6 months	Not reported	Not reported
Pooled HR			0.49 (0–37-0.65)

#### 3.4.2. Melanoma

Seven studies (n = 1164) analyzed ICIs beyond progression in melanoma. ORRs ranged from 16% to 28%, with the highest in the phase 3 trial by Long et al. (28%) and KEYNOTE-001 (24.4%). Median OS was longer for patients treated beyond progression (24–29 months) compared to those who stopped treatment (8–11 months).

#### 3.4.3. RCC

Seven studies (n = 458) evaluated RCC. ORRs ranged from 13% to 33.3%. The results support continued ICI use in select RCC patients, especially those with favorable inflammatory profiles.

#### 3.4.4. Other Cancers

Fifteen studies evaluating the efficacy of ICIs beyond progression were reviewed, encompassing CRC, head-and-neck, liver, gastric, and urothelial cancers. These studies included retrospective cohort analyses, post hoc analyses, a phase 3 trial (gastric cancer), and one phase 2 trial. Across the analyzed studies investigating the use of ICIs beyond progression in other solid tumors, considerable heterogeneity in clinical outcomes was observed. Objective response rates varied substantially, ranging from 0% in Parseghian et al. (colorectal and pancreatic cancers) to 47.6% in Fukuokaya et al. (urothelial cancer). Median PFS was reported in a subset of studies, with the longest PFS observed in Bei et al. (8.4 months) and Jiang et al. (7.9 months), and shorter PFS seen in gastric cancer studies such as that by Boku et al. (1.6 months). Median OS also varied across studies, ranging from 5.3 months in Boku et al. (gastric cancer) to 28.3 months in Bei et al. (head-and-neck cancer). Overall, these findings highlight the variable but potentially durable benefit of continuing ICIs beyond progression across different tumor types.

### 3.5. Safety

Safety was explicitly reported in 38 of the 50 included studies (76%), covering 7292 patients. Across tumor types, the pattern and frequency of immune-related adverse events (irAEs) after TBP closely resembled those seen during the initial course of immunotherapy:

With respect to overall incidence, any-grade irAEs were observed in 17–58% of patients, whereas grade ≥3 events rarely exceeded one-quarter of the cohort in any series. In the lung cancer literature (29 studies, 3768 pts), the proportion of grade ≥3 irAEs ranged from 10% to 25%. Similar upper limits (approximately 20–22%) were described in the largest melanoma and RCC datasets, although denominators and grading scales were inconsistently reported.

With respect to the spectrum of toxicity, the organ distribution mirrored that of first-line ICI therapy:

- Dermatologic (rash, pruritus) and endocrine (thyroiditis, hypophysitis) events were most frequent;
- Gastrointestinal (colitis, hepatitis) and pulmonary (pneumonitis) irAEs accounted for most grade ≥3 cases;
- Lateonset but serious toxicities, such as myocarditis and nephritis, were mentioned in isolated reports but remained uncommon (<1%).

In terms of comparative risk, none of the prospective or retrospective series detected a higher incidence of de novo grade ≥3 irAEs after progression than before it; several authors explicitly state that “continuation of PD-1/PD-L1 blockade does not precipitate a disproportionate incidence of severe irAEs.” Where a control group that discontinued ICIs was available (e.g., OAK and CheckMate-025 post hoc analyses), cumulative toxicity curves were superimposable.

In terms of treatment disruption, permanent ICI discontinuation for toxicity occurred in 4–9% of patients and was driven mainly by grade 3–4 colitis or pneumonitis; temporary holds with corticosteroid tapers were more common. Fatal irAEs were exceedingly rare (≤0.5%), with single-case reports of fulminant myocarditis in NSCLC and hepatic failure in melanoma cohorts.

With respect to management considerations, most series emphasized the need for heightened vigilance for delayed irAEs, as median on-treatment exposure during TBP exceeded six months in responding patients. Re-escalation of checkpoint therapy after a steroid taper was feasible in about one-half of cases, provided that the index event resolved to ≤grade 1.

### 3.6. Biomarkers and Predictive Factors

Predictors of benefit from ICIs beyond progression included performance status (ECOG 0–1), early response (PR/SD), oligoprogression, and low systemic inflammation (e.g., mGPS in NSCLC, CRP in RCC). MSI-H status in CRC also indicated benefit. Continuation with the same PD-1 inhibitor was more effective than switching. Poorer outcomes were linked to high tumor burden, new metastases, or poor performance. In MSS CRC and pancreatic cancers, pseudoprogression was rare; TBP was generally ineffective. Although only a minority of the included studies reported genomic data, two post hoc analyses and one large, real-world cohort consistently indicated that a higher baseline TMB enriches for benefit when ICIs are continued past RECIST progression. Mechanistically, a high TMB increases neo-antigen load and the likelihood of maintaining immunogenic subclones despite clonal evolution at progression, thereby sustaining the benefit of continued PD-(L)1 blockade. Conversely, a very low TMB may indicate primary immune-ignorant biology, where TBP is unlikely to help.

### 3.7. Meta-Analysis of TBP in Cancer

#### 3.7.1. Response Rates to Treatment Beyond Progression

- Overall response rate (ORR): 16.2% (95% CI: 12.8–20.1%);
- Disease control rate (DCR): 67.5% (95% CI: 61.3–73.2%).

#### 3.7.2. Other Cancers (Table 4)

- RCC shows particularly strong benefit (HRs 0.18–0.54);
- HCC benefits from multiple post-progression strategies, with combined approaches (ICI + TKI) showing the greatest benefits;
- Benefits observed across various tumor types in mixed-cancer studies.

**Table 4:** Other cancers: OS and PFS.

Study	Cancer Type	Outcome	Hazard Ratio (95% CI)	*p*-Value
George et al. [49]	mRCC	OS	Not directly reported	-
Talbot et al. [59]	HCC	PPS (ICI beyond progression)	0.52 (0.32–0.84)	0.0075
Talbot et al. [59]	HCC	PPS (Post-PD TKI)	0.38 (0.25–0.56)	<0.0001
Talbot et al. [59]	HCC	PPS (ICI + subsequent TKI)	0.24 (0.12–0.49)	0.0001
Talbot et al. [59]	HCC	PPS (Other post-PD therapies)	0.41 (0.22–0.73)	0.0031
Guven et al. [24]	Various	OS (TBP vs. non TBP)	0.50 (0.35–0.72)	<0.001
Lim et al. [57]	HCC	PFS2 and OS2	Comparable outcomes	-
Saal et al. [29]	RCC	OS (TBP in low-risk)	0.18 (0.06–0.55)	0.002
Saal et al. [29]	UC	OS (TBP in low-risk)	0.59 (0.34–1.00)	0.052
Murianni et al. [64]	mRCC	OS (TBP vs. non-TBP)	0.54 (0.40–0.72)	<0.001

#### 3.7.3. Overall Pooled Analysis (All Studies; Table 5)

The meta-analysis demonstrates a consistent benefit for TBP across both lung cancers and other cancer types. The overall pooled hazard ratio of 0.53 (95% CI: 0.47–0.60) indicates a 47% reduction in the risk of death for patients receiving TBP compared to those who discontinued treatment at progression.

**Table 5:** Overall pooled analysis (all studies).

Subgroup	Hazard Ratio (95% CI)	Notes
**By Cancer Type**		
–Lung cancers	0.53 (0.44–0.64)	Consistent OS benefit
–Other cancers	0.52 (0.42–0.65)	No difference vs. lung
–Comparison (lung vs. other)	—	*p* = 0.91 (NS)
**By Treatment Type**		
–Immunotherapy beyond progression	0.45 (0.36–0.57)	Larger OS benefit
–Other TBP approaches	0.59 (0.51–0.69)	Benefit but less pronounced
–Comparison (ICI vs. other TBP)	—	*p* = 0.07 (trend only)

#### 3.7.4. Heterogeneity Assessment

- Moderate heterogeneity was observed in the lung cancer studies (I^2^ = 67%), likely reflecting differences in patient populations, treatment regimens, and study designs;
- Low heterogeneity was observed in the non-lung cancer studies (I^2^ = 0%);
- The overall heterogeneity was moderate (I^2^ = 52%).

### 3.8. Ratings of the Quality of Evidence

Overall, with respect to the GRADE of Recommendation (Supplementary Table S6), the evidence supporting the clinical efficacy and safety of ICIs beyond radiological progression ranges from low to high quality depending on cancer type, with melanoma and gastric cancers presenting the most robust evidence base due to the inclusion of randomized controlled trials. Moderate-quality evidence supports continued ICI use in lung cancer, renal cell carcinoma, and urothelial cancer, while the evidence for colorectal, head-and-neck, and liver cancers remains limited by study design and variability in patient selection. This structured grading provides clinicians and researchers with clear guidance on the confidence and applicability of current evidence when considering the continuation of ICIs beyond progression in clinical practice.

## 4. Discussion

The systematic review and meta-analysis indicate that the continuation of ICIs beyond radiological progression—commonly termed TBP—can yield meaningful clinical benefits in carefully selected patients with advanced malignancies. The most consistent evidence is seen in melanoma and NSCLC, where randomized trials and large retrospective cohorts have demonstrated improvements in OS and PFS compared with discontinuation. Similar, though less robust, benefits have been reported in small cell lung cancer, renal cell carcinoma, and urothelial carcinoma, while evidence in colorectal, head-and-neck, and hepatocellular cancers remains limited and heterogeneous, underscoring the need for prospective validation [4,10,11,12,13,65,66,67,68,69].

The rationale for TBP differs from that of chemotherapy continuation in indolent tumors. Whereas cytotoxic drugs primarily maintain cytostatic pressure, ICIs aim to sustain or rekindle immune responses capable of long-term tumor control [70]. Durable benefits observed in melanoma, NSCLC, and RCC suggest that ongoing PD-(L)1 blockade can preserve immunologic memory and counteract resistant subclones, thereby distinguishing immunotherapy from earlier paradigms of treatment continuation.

Clinically, TBP appears most appropriate for patients who previously derived benefit from ICIs and subsequently experienced indolent or oligoprogressive disease. Observational data support combining local ablative strategies (e.g., surgery or stereotactic radiotherapy) with continued systemic therapy in these scenarios, thereby prolonging disease control without the need for immediate systemic switch. Conversely, multifocal or rapidly progressive disease is less likely to benefit, highlighting the importance of careful patient selection.

An accurate distinction between true progression and atypical immune-related dynamics (such as pseudoprogression) is pivotal. Immune-adapted criteria (iRECIST) and short-interval re-imaging can prevent premature discontinuation, while emerging tools, such as circulating tumor DNA kinetics, FDG-PET, and radiomics, may provide complementary information. Integrating these biomarkers prospectively could refine candidate selection for TBP and minimize unnecessary exposure [71,72].

Although the safety profile of TBP closely mirrors that of first-line ICI therapy, rechallenge after prior high-grade immune-related adverse events carries higher risks, with recurrence observed in approximately one-third of cases. Nevertheless, durable responses in a subset of these patients support its consideration when therapeutic alternatives are limited. Together, these findings argue for a nuanced, biomarker-informed approach to TBP, balancing the potential for extended benefit against the risks of toxicity and resource use.

An accurate determination of true versus false progression is pivotal. Immune-adapted criteria, such as iRECIST, should confirm progression; clinically stable patients ought to be re-imaged after four to eight weeks to exclude pseudoprogression. Advanced imaging (for example, FDG-PET) and radiomic artificial intelligence models can identify lymphocytic infiltration or necrosis that masquerade as enlargement on CT. Rising ctDNA levels generally signal true progression, whereas stable or falling levels suggest a flare phenomenon. The persistence of PD-1^high CD8^+ T cells, a strong interferon-γ signature, and broad TCR diversity further support ongoing immune activity. Integrating these tools prospectively should improve TBP stewardship and study design [73,74,75,76].

When tumors escape checkpoint control, the layering of complementary mechanisms can restore sensitivity. Conventional chemotherapy induces immunogenic cell death, supplies neoantigens, and depletes myeloid-derived suppressor cells; the combining of platinum doublets with PD-1/PD-L1 blockade in pre-treated NSCLC or urothelial cancer has raised response rates to the 20–30% range [77,78,79,80]. PARP inhibition in BRCA-mutated tumors activates cGAS–STING signaling and enhances antigen presentation, yielding objective responses exceeding 40% in early-phase trials when paired with PD-1 antibodies [81,82]. Next-generation partners—including TIGIT- or LAG-3-blocking antibodies, CD47 inhibitors, and oncolytic viruses—aim to broaden the immune attack through both innate and adaptive arms.

It is equally important to distinguish how and when patients are being re-exposed to checkpoint inhibition, because the clinical logic and risk–benefit calculus differ markedly between the two main scenarios. In treatment beyond radiologic progression, therapy is carried straight through the first RECIST or iRECIST progression in clinically stable patients. The evidence base—dominated by retrospective lung cancer, melanoma, and renal cell series—indicates that the benefits concentrate in the biologically favorable subgroup we identified earlier: those who enjoyed an initial response, experience only oligoprogression, and remain in ECOG 0–1 condition. Safety profiles during TBP resemble those observed during first-line treatment: new grade ≥3 immune-related adverse events (irAEs) are not disproportionately increased. Rechallenge after a prior high-grade irAE carries greater risk—about one-third of patients experience recurrence of the same toxicity and 10–15% develop a new event; however, roughly 20% still achieve an objective response, supporting the practice when therapeutic alternatives are limited and toxicity has resolved.

A critical evaluation of the included studies reveals considerable heterogeneity in patient selection, treatment protocols, and outcome reporting. The predominance of retrospective cohorts (82%) introduces substantial selection bias and limits causal inference, while publication bias and potential patient overlap in large registries may further distort pooled estimates. Clinical heterogeneity was pronounced: patients differed in disease setting (first-line vs. later-line); progression burden (oligo- vs. multifocal); prior locoregional therapy; checkpoint backbone (PD-1/PD-L1 monotherapy, CTLA-4 combinations, or sequential blockade); and progression criteria (RECIST 1.1, iRECIST, or investigator judgment). Between-study inconsistency was likewise methodological, with sample sizes ranging from 25 to 2495, follow-up times seldom exceeding 12 months, and mostly unadjusted statistical approaches (I^2^ = 67% for overall survival). Nonetheless, the consistent clinical benefits observed—particularly in melanoma, NSCLC, RCC, gastric cancer, and, to a lesser extent, SCLC—suggest that treatment continuation beyond progression warrants consideration in carefully selected patients. Emerging biomarkers, such as the modified Glasgow Prognostic Score in lung cancer, along with PD-L1 expression and ctDNA kinetics, may help to refine candidate selection, although they require prospective validation. Meticulous monitoring, preferably within prospective cohorts or biomarker-stratified trials, remains essential to confirm the durability of the benefit and to mitigate late toxicities.

However, the findings of this systematic review are broadly concordant with real-world evidence derived from large clinical registries already included in this review, which likewise report the modest but durable benefits of continuing ICIs beyond RECIST progression in well-selected patients. For example, registry-based studies in NSCLC and melanoma have confirmed that patients with good performance status, prior clinical benefit, and oligoprogression can achieve prolonged disease control and overall survival when ICIs are continued. These registries also reinforce the safety signals observed in our review, showing no major increase in immune-related adverse events during treatment beyond progression. However, clinical registries often lack uniform progression criteria, radiologic adjudication, and biomarker data, which may obscure the contribution of pseudoprogression or immune-mediated dynamics. Moreover, registries rarely capture ctDNA kinetics or immune signatures that might further refine patient selection. These limitations underscore the added value of integrating registry data with prospective biomarker-driven studies to guide treatment beyond progression.

Guideline positions differ. The National Comprehensive Cancer Network (NCCN) endorses cautious TBP for melanoma and NSCLC [82,83], whereas the European Society for Medical Oncology and the Society for Immunotherapy of Cancer remain less prescriptive, reflecting ongoing uncertainty [84,85,86,87,88]. From a health-economics perspective, TBP becomes cost-effective when the probability of durable benefit exceeds 20% or the time to treatment failure extends beyond ten months. Biomarker-based enrichment could help meet these thresholds by sparing non-responders the toxicity and expense of prolonged therapy.

Several research priorities emerge. Prospective, biomarker-stratified randomized trials comparing TBP with immediate switch therapy are urgently needed. Standardizing progression criteria and embedding ctDNA and advanced imaging into response assessments will improve comparability. Quality-of-life and cost–utility endpoints should accompany clinical outcomes; the exploration of adaptive or intermittent dosing may mitigate cumulative toxicity. Finally, evaluating novel checkpoint and co-stimulatory combinations in post-progression settings, supported by multi-omics biobanking and real-world data integration, should refine predictive algorithms.

In daily practice, continuing or rechallenging PD-(L)1 blockade should be reserved for patients who derived clear initial benefits, display oligoprogression that can be locally ablated, and retain ECOG 0–1 status with low systemic inflammation. In this favorable subset, TBP yields response rates of 20–30% and extends median survival by six to twelve months, with toxicity comparable to first-line therapy. Conversely, primary non-responders, those with multifocal visceral relapse, or individuals with unresolved grade ≥2 cardiopulmonary or neurological irAEs should transition to alternative systemic therapy or enter trials that combine non-redundant mechanisms, such as platinum chemotherapy or PARP inhibition, to overcome adaptive resistance.

In conclusion, immune checkpoint blockade beyond conventional RECIST-defined progression is a rational strategy for well-selected patients; however, the current evidence is predominantly retrospective and methodologically heterogeneous. Harmonizing progression definitions, prospectively validating biomarkers, and conducting biomarker-enriched randomized trials are essential steps toward translating the immunobiological rationale of TBP into consistent clinical benefits.

## Supplementary Materials

The following supporting information can be downloaded at: https://www.mdpi.com/article/10.3390/jcm14186680/s1, Table S1: Characteristics of included studies; Table S2: Summary of lung cancer studies; Table S3: Summary of melanoma studies; Table S4: Summary of renal cell carcinoma; Table S5: Summary of other cancers studies; Table S6: GRADE of evidence.
