# Supplementary material for: Immune Checkpoint Inhibitors Beyond Progression in Various Solid Tumors: A Systematic Review and Pooled Analysis
Source: J Clin Med. 2025 Sep 22;14(18):6680. doi: 10.3390/jcm14186680 (PMC12470277; doi:10.3390/jcm14186680)
Supplement: Supplementary file 1 [file jcm-14-06680-s001.zip › jcm-3786960-supplementary.pdf]

**Table S1.** Characteristics of included studies

| Study (year) - Cancer type | Patients (n) | Study Type | ORR % | Median PFS (months) | Median OS (months) | Quality (NOS score) | Prognostic factors |
|---|---|---|---|---|---|---|---|
| **Chen (2023) - NSCLC and SCLC** | 99 | Retrospective | 12.1 | 0.62 (HR: 0.40-0.95) | 14.1 (HR: 0.48, 95CI 0.40-0.95) | 6 | Age, gender,<br>smoking status, PS, therapy line,<br>RT, lung, bone and node metastasis |
| **Cheng Y (2024) - NSCLC** | 93 | Retrospective | 34.4 | 5.5<br>(HR: 1.59, 95CI 1.15-2.20) | 13.3 (HR: 1.65, 95CI 1.08-2.50) | 7 | NLR, PS, choice of second-line treatment |
| **Cheng J (2025) - NSCLC and SCLC** | 78<br><br><br>31 | Retrospective | 35.9<br><br><br>38.7 | 4.4 (HR: 0.7, 95CI 0.42-1.17)<br>3.9 (HR: 0.38, 95CI 0.16-0.87) | 10.3 (HR: 1.34, 95CI 0.49-3.70)<br>9.2 (HR: 0.23, 95CI 0.08-1.77) | 7 | - |
| **Deng (2024) - NSCLC** | 50 | Retrospective | - | 32.1* (HR: 0.50, 95CI 0.22-1.16) | - | 7 | PS, antiangiogenesis therapy |
| **Enomoto (2021) - NSCLC** | 28 | Retrospective | 25 | 12.2* (HR: 0.64, 95CI 0.31-1.31) | 15.6 (HR: 0.78, 95CI 0.44-1.39) | 6 | Smoking history, CRP, ALI, response PR or SD |
| **Gandara (2018) - NSCLC** | 168 | Phase 3 | 16 | 4.2 | 8.6 | 8 | - |
| **Ge (2020) - NSCLC** | 39 | Retrospective | 15.4 | 8.9 (HR: 0.41, 95CI 0.26-0.65) | 26.6 (HR: 0.4, 95CI 0.23-0.69) | 6 | Gender, squamous histology, no brain or liver M+, not beyond ≥ 3rd line, with PR to the previous ICI and monotherapy as previous ICI |
| **Guven (2023) - NSCLC** | 82 | Retrospective | - | 13.1* (HR: 0.50, 95CI 0.34-0.71) | - | 7 | - |
| **Li (2022) - SCLC** | 45 | Retrospective | 26.7 | 4.8 (HR: 0.40 (0.24-0.67) | 17.4 (HR: 0.55, 95 0.29-1.04) | 6 | Gender, first-line initial IO, initial PD-L1 inhibitors, SD/PD response to initial IO, no brain or liver metastases and ECOG 0–1 |
| **Liang (2020) - NSCLC** | 10 | Retrospective | - | - | - (HR: 2.8, 95CI 2.7-13.6) | 5 | - |
| **Ozguroglu (2023) - NSCLC** | 64 | Retrospective | 20 | 6.6 | 15.1 | 6 | - |
| **Pourmir (2023) - NSCLC** | 43 | Retrospective | - | 21.3* (HR: 0.14, 95CI 0.07-0.30) | - | 6 | - |
| **Ricciuti (2019) - NSCLC** | 60 | Retrospective | 46.6 | 3.5 | 17.8 (HR: 0.32, 95CI 0.21-0.46) | 7 | - |
| **Saal (2025) - NSCLC** | 1071 | Post hoc analysis of phase 3 | - | - | - | 8 | - |
| **Salous (2022) - NSCLC** | 35 | Phase 2 | 23.5 | 5.1 | 24.5 | 6 | - |
| **Shi (2025) - SCLC** | 28 | Retrospective | - | 4 | 18.8 | 6 | primary liver metastasis, 1L treatment response, progression free<br>interval and depth of response during 1L chemo-IO |
| **Singhi (2023) - NSCLC** | 33 | Retrospective | - | 4.1 | 31.5 | 6 | - |
| **Stinchombe (2020) - NSCLC** | 1668 | Retrospective | - | - | 11.5 | 9 | - |

| | | | | | | | |
|---|---|---|---|---|---|---|---|
| **Tian (2021) - NSCLC** | 92 | Retrospective | 11.2 | 3.9 | 11.8 | 7 | PS 0-1, controlled metastases |
| **Topp (2023) - NSCLC** | 94 | Post hoc analysis of phase 3 | 17.0 | 4.5 | 15.0 | 8 | ICI BP response |
| **Wang C (2025) - NSCLC** | 112 | | 14.3 | 3.7 | 11.6 | 7 | Prior ICI response, PS 0–1 |
| **Wang M (2024) - NSCLC** | 104 | Retrospective | 15.7 | 4.0 | 13.0 | 7 | Response duration >6 , better PS ECOG |
| **Wang Y (2023) - NSCLC** | 148 | Retrospective | 12.8 | 5.5 | 18.0 | 7 | Response to 1st line, no new lesions |
| **Won (2020) - NSCLC** | 59 | Retrospective | - | 4.1 | 14.9 | 7 | 1st line response |
| **Xu M (2023) - NSCLC** | 173 | Retrospective | - | 4.2 | 13.2 | 7 | 1st line PR/SD, interval chemo benefit, PS ≤1 |
| **Xu Y (2021) - NSCLC** | 109 | Retrospective | 10.7 | 4.3 | 15.7 | 7 | Oligoprogression, 1L PFS ≥6 |
| **Yamamoto (2024) - SCLC** | 108 | Retrospective | 12.0 | 3.9 | 10.3 | 7 | PR/SD at 1L, early stabilization |
| **Yan (2024) - NSCLC** | 224 | Retrospective | 10.3 | 5.33 | - | 8 | Response to 1st line ICI, not resistance, better PS ECOG |
| **Yin (2025) - NSCLC** | 154 | Retrospective | 16.4 | 4.3 (HR: 0.66, 95CI 0.11-0.95) | - | 7 | Response to initial IO, PFS1, IO combined with TT or switch to a different IO |
| **Ahmed (2021)- MELANOMA** | 78 | Retrospective | 17.9** | - | 8 | 7 | Prior response, pseudoPD |
| **Beaver (2018)- MELANOMA** | 692 | FDA pooled analysis | 19 | - | 24.4 | 8 | PS, LDH, tumor burden at PD |
| **Czarnecka (2022)- MELANOMA** | 77 | Retrospective | 23.4 | 8.02 | 28.75 | 7 | Type/site of M+ |
| **Guven (2023)- MELANOMA** | 19 | Retrospective | - | - | - (HR: 0.5,95 CI: 0.34–0.71), | 6 | PS |
| **Long (2017)- MELANOMA** | 85 | Retrospective analysis of a Phase 3 trial | 28 | 4.7 | - | 8 | Tumor burden at PD, type/site of M+ |
| **Topp (2023)- MELANOMA** | 176 | Post-hoc analysis of KEYNOTE 001 | 24.4 | - | - | 7 | - |
| **Zimmer (2017)- MELANOMA** | 37 | Retrospective° | 16 | - | - | 6 | PS, LDH, prior response |
| **Escudier (2023)- RCC** | 153 | Retrospective analysis of Checkmate 025 | 13 | - | 20.4 | 8 | - |
| **George (2023)- RCC** | 36 | Retrospective analysis of a phase II study | 33 | - | 22.5 | 5 | - |
| **Ishihara (2020)- RCC** | 17 | Retrospective | 24 | 7.53 | Not reached | 5 | Reactive C protein |
| **Murianni (2024)- RCC** | 93 | Retrospective | 33.3 | 6.7 | 34.8 (HR: 0.54, 95 CI 0.40–0.72) | 8 | TBP |
| **Saal (2024)- RCC** | 148 | Post-hoc analysis of IMmotion 151 trial | - | - | - | 8 | mGPS |
| **Topp (2023)-RCC** | 51 | Post-hoc analysis of KEYNOTE 427 trial | 15.7 | - | - | 8 | - |
| **Guven (2023)-RCC** | 68 | Retrospective | - | 5.69 | - (HR: 0.5, 95 CI: 0.34–0.71) | 7 | - |
| **Colle (2023) - CRC** | 29 | Retrospective | 10 | - | - | 6 | Previous clinical benefit, PS 0-1, pseudoprogression |

| | | | | | | | |
|---|---|---|---|---|---|---|---|
| **Parseghian (2023) – CRC and pancreatic** | 59 | Retrospective | 0 | - | 10.2 (HR: 0.5, 95 CI: 0.3–0.8) | 6 | PS 0-1, Rapid tumor growth or new lesions at PD, low marker increase |
| **Bei (2023) - Head & Neck** | 37 | Retrospective | 37.8 | 8.4 (HR:0.54,95CI 0.32-0.94) | 28.3 (HR: 0.39,95CI 0.13-1.17) | 7 | - |
| **Jiang (2023) - Head &** Neck | 48 | Retrospective | 31.2 | 7.9 | - | 6 | - |
| **Haddad (2023) - Head & Neck** | 62 | Retrospective | 16 | 3.7 | 12.7 | 6 | - |
| **Talbot (2023) - Liver** | 364 | Retrospective | - | - (HR:0.52, 95CI 0.32–0.84) | 5.3 (HR: 0.5, 95CI 0.27-0.92) | 7 | - |
| **Chen C (2023) - Liver** | 113 | Retrospective | - | - | 8.6 | 8 | - |
| **Lim M (2023) - Liver** | 34 | Retrospective | 43.8 | 3.7 | 17 | 6 | - |
| **Ren (2023) - Liver** | 102 | Phase 2 | 23.5 | - | 16.9 | - | - |
| **Boku (2021) - Gastric** | 330 | Phase 3 | 11.2 | 1.6 | 5.3 (HR: 0.69, 95CI 0.47–1.01) | - | - |
| **Li (2024) - Gastric** | 80 | Retrospective | 7.1 | 5.4 | 10.4 (HR:0.46, 95CI 0.26-0.81) | 7 | PS 0, ICI beyond PD, first-line tx, previous clinical benefit |
| **Fukuokaya (2022) - Urothelial** | 21 | Retrospective | 47.6 | - | 17.8 (HR: 0.21, 95CI 0.05-0.9) | 6 | ICI beyond PD |
| **Saal (2024) – (urothelial)** | 176 | Post-hoc analysis | - | - | - | 8 | Low mGPS favorable prognostic factor |
| **Topp (2023) - Urothelial** | 227 | Post-hoc analysis | 13.2 | - | - | 8 | Early shrinkage or stabilization |
| **Topp (2023) - Gastric** | 87 | Post-hoc analysis | 12.6 | - | - | 8 | Early shrinkage or stabilization |
| **Topp (2023) - Head & Neck** | 112 | Post-hoc analysis | 8.9 | - | - | 8 | Early shrinkage or stabilization |
| **Necchi (2017) - Urothelial** | 137 | Post-hoc analysis | 11.7 | 2.1 | 8.6 | 7 | PS 0, non visceral M+ |

***, post progression survival; ORR, overall response rate; PFS, progression-free survival; OS, overall survival; HR hazard ratio; NSCLC, non-small-cell lung cancer; SCLC, small-cell lung cancer; Retrosp, retrospective; Random, randomized; PS, performance status; RT, radiotherapy; NLR, neutrophil-to-lymphocyte ratio; CRP, c-reactive protein; ALI, advanced lung cancer inflammation index; PR, partial response; SD, stable disease; IO, immunotherapy; -, not reached; **pseudoprogression; °, ipilimumab + nivolumab arm**

**Table S2.** Summary of lung cancer studies

| Characteristic | Data Summary |
|---|---|
| **Total Studies** | 29 studies across NSCLC and SCLC |
| **Total Patients** | 3.768 |
| **Study Types** | Real-world retrospective studies, post-hoc analyses of RCTs, phase II trials |
| **Tumor Types** | Predominantly NSCLC, with 3–4 SCLC-focused studies |
| **Quality (Newcastle-Ottawa Score, NOS)** | Scores ranged from 5 to 9 |
| **Overall Response Rate** | Range: 2.4%–28%; up to 42.9% in selected rechallenge responders |
| **Median PFS** | Reported in most studies; typically 2.9–9.7 months |
| **Median OS** | Varies widely: ~7.4–28.8 months (longer with TBP, rechallenge or combo) |
| **Notable Prognostic Factors** | Favorable: oligoprogression, prior PR/SD, local radiotherapy, new ICI class, antiangiogenic combo |

**Table S3.** Summary of melanoma studies

| Characteristic | Data Summary |
|---|---|
| **Total Studies** | 7 |
| **Total Patients** | 1164 |
| **Study Types** | Mostly retrospective studies (6/7); 1 FDA pooled analysis; 1 post-hoc analysis of a Phase 1 trial |
| **Overall Response Rate** | Range: 16% to 28% |
| **Median PFS** | Reported in 3 studies, range: 4.7 to 8.02 months |
| **Median OS** | Reported in 4 studies, range: 8 months to 28.75 months |
| **Quality (Newcastle-Ottawa Score, NOS)** | Scores ranged from 6 to 8 |
| **Common Prognostic Factors Identified** | Performance status (PS), LDH levels, tumor burden at PD, prior response, type/site of metastases (M+), presence of pseudoprogression |

**Table S4.** Summary of renal cell carcinoma

| Characteristic | Data Summary |
|---|---|
| **Total Studies** | 7 |
| **Total Patients** | 566 |
| **Study Types** | Retrospective and post-hoc analyses |
| **Overall Response Rate** | Range: 13% to 33.3% (reported in 5 studies) |
| **Median PFS** | Reported in 3 studies, range: 5.69 to 7.53 months |
| **Median OS** | Reported in 3 studies, range: 20.4 to 34.8 months |
| **Quality (Newcastle-Ottawa Score, NOS)** | Scores ranged from 5 to 8 |
| **Common Prognostic Factors Identified** | Reactive C protein, treatment beyond progression, modified Glasgow Prognostic Score |

**Table S5.** Summary of other cancers studies

| Characteristic | Data Summary |
|---|---|
| **Total Studies** | 17 |
| **Total Patients** | 2157 |
| **Study Types** | Mostly retrospective and post-hoc analyses; includes 2 phase trials |
| **Overall Response Rate** | Range: 0% to 47.6% (reported in 13 studies) |
| **Median PFS** | Reported in 6 studies, range: 1.6 to 8.4 months |
| **Median OS** | Reported in 10 studies, range: 5.3 to 28.3 months |
| **Quality (Newcastle-Ottawa Score, NOS)** | Scores ranged from 5 to 8 |
| **Common Prognostic Factors Identified** | PS 0–1, ICI beyond PD, pseudoprogression, early shrinkage or stabilization, low mGPS |

**Table S6.** GRADE of evidence

| Cancer Type | Number of Studies | Total Patients | Study Designs | Quality of Evidence | GRADE Rating |
|---|---|---|---|---|---|
| **Lung Cancer (NSCLC/SCLC)** | 29 | 3,768 | Mostly retrospective cohorts, few post-hoc analyses | Moderate | ⊕⊕⊕◯ |
| **Melanoma** | 7 | 1164 | Retrospective cohorts, FDA pooled analysis, phase 3 trial | Moderate to High | ⊕⊕⊕◯ / ⊕⊕⊕⊕ |
| **Renal Cell Carcinoma** | 7 | 566 | Retrospective cohorts, post-hoc analyses | Moderate | ⊕⊕⊕◯ |
| **Colorectal Cancer** | 2 | 88 | Retrospective cohorts | Low | ⊕⊕◯◯ |
| **Head & Neck Cancer** | 4 | 259 | Mostly retrospective cohorts, one post-hoc analysis, one case series | Low to Moderate | ⊕⊕◯◯ / ⊕⊕⊕◯ |
| **Liver Cancer** | 4 | 613 | Retrospective cohorts | Low | ⊕⊕◯◯ |
| **Gastric Cancer** | 3 | 497 | Phase 2/3 trials, retrospective cohort, post-hoc analyses | Moderate to High | ⊕⊕⊕◯ / ⊕⊕⊕⊕ |
| **Urothelial Cancer** | 4 | 186 | Retrospective cohorts, post-hoc analyses | Moderate | ⊕⊕⊕◯ |

**Interpretation of GRADE Ratings**

- **⊕⊕⊕⊕ (High Quality)**:
  Very confident that the true effect closely matches the estimated effect. Example: Melanoma and Gastric cancer evidence derived from prospective phase 3 trials.
- **⊕⊕⊕◯ (Moderate Quality)**:
  Moderately confident in the effect estimate; the true effect is likely close to the estimate but could substantially differ. Example: Lung Cancer (NSCLC and SCLC), Renal Cell Carcinoma, and Urothelial cancer studies with predominantly retrospective studies and post-hoc analyses.
- **⊕⊕◯◯ (Low Quality)**:
  Limited confidence in the effect estimate; the true effect might substantially differ from the estimate. Example: Colorectal, Head & Neck, and Liver cancers due to primarily retrospective cohort studies with inherent methodological limitations and heterogeneity.
- **⊕◯◯◯ (Very Low Quality)**:
  Little confidence in the effect estimate; the true effect is likely significantly different from the estimate.
